# Psychiatric Symptoms in Amyotrophic Lateral Sclerosis: Beyond a Motor Neuron Disorder

**DOI:** 10.3389/fnins.2019.00175

**Published:** 2019-03-11

**Authors:** Elisabetta Zucchi, Nicola Ticozzi, Jessica Mandrioli

**Affiliations:** ^1^Department of Neuroscience, Azienda Ospedaliero Universitaria di Modena, University of Modena and Reggio Emilia, Modena, Italy; ^2^Department of Neurology and Laboratory of Neuroscience, Istituto Auxologico Italiano, Istituto di Ricovero e Cura a Carattere Scientifico, Milan, Italy; ^3^Department of Pathophysiology and Transplantation, ‘Dino Ferrari’ Center, Università degli Studi di Milano, Milan, Italy; ^4^Department of Neuroscience, Azienda Ospedaliera Universitaria Modena, St. Agostino- Estense Hospital, Modena, Italy

**Keywords:** amyotrophic lateral sclerosis, frontotemporal dementia, psychosis, depression, c9orf72, psychiatric symptoms and disorders

## Abstract

The historical view that Amyotrophic Lateral Sclerosis (ALS) as a pure motor disorder has been increasingly challenged by the discovery of cognitive and behavioral changes in the spectrum of Frontotemporal Dementia (FTD). Less recognized and still significant comorbidities that ALS patients may present are prior or concomitant psychiatric illness, such as psychosis and schizophrenia, or mood disorders. These non-motor symptoms disturbances have a close time relationship with disease onset, may constitute part of a larger framework of network disruption in motor neuron disorders, and may impact ALS patients and families, with regards to ethical choices and end-of-life decisions. This review aims at identifying the most common psychiatric alterations related to ALS and its prognosis, looking at a common genetic background and shared structural brain pathology.

## Introduction: Amyotrophic Lateral Sclerosis and Its Phenotypes

Amyotrophic Lateral Sclerosis (ALS) has traditionally been defined since the first reports as a disorder characterized by progressive degeneration of upper and lower motor neurons (UMN and LMN, respectively), leading invariably to paralysis of voluntary muscles, with a variable proportion of spasticity and atrophy. Despite a uniformly fatal outcome, extreme variability exists within ALS, with heterogeneity of initial presentation, spreading of disease, progression, and survival (Brown and Al-Chalabi, 2017; Hardiman et al., 2017). The observation of distinct patterns within ALS clinical variability has led to the recognition of homogeneous phenotypic subgroups. A first classification system is based on the differential involvement of upper and lower motor neuron, with primary lateral sclerosis (PLS) and progressing muscular atrophy (PMA) representing the extremes of the spectrum. The type of involvement of different body regions at onset is another common identifier, with bulbar patients constituting 25% of the total ALS population (Chiò et al., 2011). This phenotype is more consistently associated with cognitive alterations and displays decreased survival compared to the classic spinal-onset, “Charcot-type” phenotype (Chiò et al., 2011; Talman et al., 2016). A prevalent LMN involvement define flail arm and flail leg variants (Wijesekera et al., 2009), whereas a prevalence of UMN signs with spasticity, increased and pathological reflexes and pseudobulbar affect, identify the UMN-predominant phenotype (UMN-p); these phenotypes display a relatively long survival (Sabatelli et al., 2008; Chiò et al., 2011). Lastly, the respiratory phenotype, characterized by an early and prominent involvement of the respiratory muscles, is characterized by the worst prognosis (Shoesmith et al., 2007). Moreover, while the majority of patients report a pattern of spreading of the disease from one limb to the contralateral, as by means of contiguity in cortical representations, there is a substantial fraction of ALS population describing a close sequential involvement of two distal sites (Walhout et al., 2017).

The clinical heterogeneity of ALS is reflected at molecular level in many ways. First of all, up to 20% of ALS patients show familiality for the disease, most typically transmitted by an autosomal dominant pattern (Gibson et al., 2014; Ryan et al., 2018). Among familial ALS (fALS), two thirds of the cases can be explained by pathogenic mutations in the C9orf72, SOD1, TARDBP, and FUS genes (Zou et al., 2017; Chiò et al., 2014), which also occur in 10–15% of sporadic ALS (sALS) (Renton et al., 2014). The relative contribution of each gene mutation to the epidemiology of the disease differs according to the population origin, with C9orf72 repeat expansion representing the most frequent alteration in ALS patients of European descent, while SOD1 mutations dominate the genetic landscape of ALS in China, Korea, and Japan (Zou et al., 2017). Overall, variable penetrance, genetic pleiotropy and the finding of double pathogenic mutations in the same patient point to an oligogenic mode of inheritance for many cases (Van Blitterswijk et al., 2012). At the histopathological level, diverse pathological signatures correspond to this fragmented genetic scenario. In the majority of sALS, as well as nearly half of frontotemporal dementia (FTD) cases, ubiquitinated and phosphorylated cytosolic TDP-43 aggregates are found in the frontal cortex (Neumann et al., 2006; Braak et al., 2010), whereas motor neurons of ALS patients harboring mutations in SOD1 or FUS display either neurofilamentous hyaline conglomerate inclusions and aggregates of misfolded SOD1 or cytoplasmic inclusions immunoreactive for FUS (Shibata et al., 1996; Kwiatkowski et al., 2009). C9orf72 associated diseases are characterized by TDP-43 pathology, but also by the presence of repeat-containing RNA (Al-Sarraj et al., 2011; Ash et al., 2013). These expanded C9orf72 RNAs form nuclear foci and can sequester various RNA-binding proteins, indirectly impairing their function on nucleic acid life cycle (Gendron et al., 2013; Zu et al., 2013). In addition, C9orf72 repeat expansions produce, via non-canonical Repeat-Associated Non-ATG (RAN) translation, several dipeptide repeat proteins (DPRs) that are highly aggregation-prone, thus compromising proteostasis (Mori et al., 2013; Kumar et al., 2017). Moreover, the human C9orf72 protein has been recently shown to play a role in endosomal degradation and lysosomal homeostasis and to target stress granules (SGs) to autophagy for clearance, acting in concert with SQSTM1 (Chitiprolu et al., 2018; Corrionero and Horvitz, 2018).

The majority of ALS-associated mutations displays an extreme variability in clinical manifestations, which may present asALS-plus phenotypes in the same individuals (e.g., ALS and parkinsonism) and/or different clinical pictures in the carriers belonging to the same family (e.g., ALS, FTD, or both in C9orf72 families).

All these recent achievements in the understanding of the disease pathogenesis led to the general consensus that ALS is a multisystem disorder in which the clinical, pathological and genetic features largely overcome the boundaries of a pure motor neuron involvement.

## ALS as an Extra-Motor Disorder With Cognitive Involvement

The common notion of ALS as a disease affecting exclusively motor neurons has been initially cast into doubt by the early clinical observations of an association with FTD. In particular, clinicians observed some degrees of motor neuron diseases (MND) in FTD patients, and conversely, signs of cognitive and behavioral changes in ALS patients (Talbot et al., 1995; Neary et al., 2000).

FTD is the second most common form of early onset dementia, characteristically presenting in the fifth–sixth decade. The term FTD is used as an umbrella which encompasses a variety of clinical subtypes defined by clinical and pathological consensus criteria (Neary et al., 2000; Gorno-Tempini et al., 2011; Rascovsky et al., 2011). FTD can present as two main types, primarily affecting behavior (behavioral variant FTD, bvFTD) or language (primary progressive aphasia, PPA), the latter of which can be further divided in semantic variant (SD), non-fluent agrammatic variant (PNFA), and logopenic variant (lvPPA) (Chare et al., 2014; Finger, 2016). Although these subtypes can have very distinct neuroanatomical substrates, with time patients with bvFTD develop impairment in language functions and vice versa. Notably, ALS is most typically associated with behavioral FTD, whereas PPA variants with MND are rare (Saxon et al., 2017). In general, nearly 15% ALS patients satisfy diagnostic criteria for FTD (Raaphorst et al., 2012a,b; Phukan et al., 2012), constituting the syndrome of ALS-FTD, while larger fractions of ALS patients exhibit mild to moderate behavioral (ALSbi) and/or cognitive deficits (ALS-eci if executive impairment is present; ALS-neci if other intellectual functions are affected). An impairment of executive functions and verbal fluency has been found in 34–55% of ALS patients (Murphy et al., 2007a,b, 2016; Lillo and Hodges, 2009; Phukan et al., 2012; van Es et al., 2017), while behavioral disturbances have been observed in 14–40% of cases (Witgert et al., 2010; Phukan et al., 2012; Abrahams et al., 2014). Even more subtle cognitive and behavioral changes can be detected by recently validated batteries especially designed for screening ALS patients (Strong et al., 2017).

On the other side, almost 15% of bvFTD patients develop ALS during the course of disease, while signs of motor neuron impairment are observed in 40% of cases (Burrell et al., 2011; Bang et al., 2015). In conclusion, ALS and FTD can be regarded as the extremes of a disease continuum sharing some common histopathological and genetic background, which reflects a much extensive involvement of the sole motor neuron pathology.

The increased understanding of this diseases spectrum, has led researchers to study a variety of symptoms not classically considered part of the ALS clinical picture, the main ones being sensory and coordination impairment, pain and autonomic involvement, sleep alteration and sphincter abnormalities. More recently, psychiatric symptoms have gained attention from several points of view: their increased presence preceding or following ALS onset, their relationship with FTD, familiality, prognosis, and treatment options.

In this review we aim to examine and elaborate on the reported aspects of psychiatric features in relation to the ALS spectrum.

## Psychiatric Disturbances in ALS and FTD

Early clinical observations reported several cases in which psychiatric illnesses such as schizophrenia co-occurred in ALS patients, raising the hypothesis of a common genetic background (Howland, 1990). More recently, register-based nationwide studies have proven an higher occurrence of psychiatric illnesses both before and after ALS diagnosis. In particular, the presence of depression, neurotic disorders and history of drug abuse or dependence, was associated to an increased odds ratio (OR) for ALS; in-depth analysis revealed that a diagnosis of schizophrenia may also represent a risk factor for ALS (OR 5.0) (Turner et al., 2016). Moreover, the risk of presenting depression, a neurotic or stress-related disorder following the diagnosis appeared to be increased as well (Longinetti et al., 2017).

Along with these findings, family members of ALS patients, especially children, showed increased risk for manifesting psychiatric disturbances both before and after their relative’s diagnosis (Longinetti et al., 2017). Further strengthening this link, aggregation studies suggest neuropsychiatric illnesses and ALS cluster in families. In a population-based cohort study the relative risk of developing a neuropsychiatric condition such as schizophrenia or psychosis was significantly higher in first or second degree relatives of ALS patients (Byrne et al., 2013; O’Brien et al., 2017). Whether this can be explained by genetic pleiotropy of few variants into several kindreds or by a shared polygenic risk between psychiatric diseases and ALS spectrum remains to be determined (O’Brien et al., 2017).

Moving to FTD, psychosis is a recognized symptom, affecting 32% of patients in the largest autoptically confirmed case series, though psychiatric disturbances are not included in the diagnostic criteria (Landqvist Waldö et al., 2015). Prevalence of hallucinations in FTD cohorts varies considerably, with auditory being the most common, and delusions affect one quarter of the patients (Hall and Finger, 2015). When retrospectively evaluating clinical features in a FTD cohort that later evolved to motor neuron disorder, the presence of delusions was the best predictor of such progression, with a hazard ratio of 4.4 (Lillo et al., 2010).

### Psychosis and Schizophrenia

Even before the discovery of a genetic overlap between schizophrenia and ALS, a relation between the two diseases was already suggested by early historical studies (Meltzer and Crayton, 1974; Howland, 1990; Larner, 2008). Moreover, disturbances in motor neuron function both at central (Goode and Manning, 1988) and peripheral level (Crayton et al., 1977; Crayton and Meltzer, 1979) exist in schizophrenia. Population-based studies have long corroborated the relation between single psychotic events, as well as schizophrenia, and ALS. In particular, an increased risk of hospitalization for schizophrenia could be observed in the 5 years preceding ALS, with higher statistical significance especially 1 year before onset of motor symptoms (Turner et al., 2016; Longinetti et al., 2017). This close relationship between psychotic features and motor symptoms in ALS may underlie the prodromal nature of these extra-motor symptoms in the framework of ALS pathogenesis (Turner et al., 2016). The link between ALS and schizophrenia was further supported by a large genome-wide association study which found a substantial genetic correlation, only partially explained by pleiotropic gene variants such as c9orf72 (McLaughlin et al., 2017). As previously highlighted, increased risk for schizophrenia and single psychotic episodes is observed among kindreds of c9orf72 carriers (Devenney et al., 2018). Moreover, psychosis was the presenting symptoms in 38% of c9orf72 carriers in a FTD-motor neuron disorder cohort, with florid psychotic symptoms such as delusional psychosis, somatoform psychosis or paranoid schizophrenia, and frontotemporal atrophy or hypoperfusion were noted on neuroimaging (Snowden et al., 2012). Delusions and hallucinations in this cohort of patients were reported to be mainly negative in nature, not related to their personal life experience (Snowden et al., 2012). Single psychotic episodes were also observed in elderly patients carrying c9orf72 expansion (Devenney et al., 2018). Overall, late-onset psychosis should always raise concern for familiality with motor neuron disease and thus warrant genetic testing for c9orf72 repeat expansion (Sommerlad et al., 2014). Furthermore, Snowden and colleagues noted that, though similar in appearance, in the case of c9orf72 expansion carriers bizarre behaviors and complex motor stereotypes had a distinct trait compared to those of other FTD and FTD-MND patients. According to the authors, it might therefore be hypothesized that this background activity of delusional thinking guides and reinforces the behavioral aberrancies typical of symptomatic FTD (Snowden et al., 2012).

Some of the cognitive changes associated to ALS, such as sensory behavioral disturbances, which were found in more than half of a large ALS cohort (Gibbons et al., 2008), have also been implicated in schizophrenia network dysfunction, with hyperactivation of secondary somatosensory cortex (Rains et al., 2012) and failed integration of sensory signaling (Carter et al., 2017). Regarding the pathophysiology of schizophrenia, a plethora of putative mechanisms have been implicated so far, mostly involving cerebral metabolic abnormalities in the pre-frontal cortex, anterior cingulate, caudate nucleus, basal ganglia, thalamus, and the cerebellum (Gross-Isseroff et al., 2003). Moreover, disruption of cortical inhibitory circuits by a reduction of overall GABAergic transmission has been advocated in schizophrenia by neurophysiological studies, mainly using transcranial magnetic stimulation (Fitzgerald et al., 2002), which demonstrated a reduction of long-interval cortical inhibition (LICI) especially in prefrontal cortex (Radhu et al., 2015). This not only relates to, similarly, enhanced cortical excitability in motor neuron disease (Geevasinga et al., 2016), but also to likewise affected tracts, with prefrontal cortex involvement in ALS patients showing verbal fluency, attention and executive function impairment (Lomen-Hoerth et al., 2003; Meier et al., 2010).

### Depression and Anxiety

Exogenous depression in ALS can be partly justified by the dismal prognosis of such a diagnosis, with patients experiencing everyday continuous motor decay. Nevertheless, literature show contrasting results in terms of the prevalence of depressive disorders in ALS, partly explained by the different testing scales employed, partly by the cross-sectional or prospective nature of these studies, and partly by the emotional adjustment or concomitant cognitive symptoms affecting ALS patients. The prevalence of depression in different studies thus ranges from impressively high values as 48–75% (McElhiney et al., 2009; Körner et al., 2015; Wei et al., 2016) to as low as 0.9–12% (Ferentinos et al., 2011; Lulé et al., 2012; Rabkin et al., 2015). In a large observational study, 17% of ALS patients were diagnosed with a major depressive disorder, and more than half of them were on antidepressant medications (Thakore and Pioro, 2016). There is evidence of an increased risk of depression prior to motor symptoms in ALS patients, particularly evident 1 year before disease onset, suggesting that the mood disorder is part of the prodromal cascade (Roos et al., 2016; Turner et al., 2016; Longinetti et al., 2017). Importantly, patients receiving a diagnosis of depression have a 3.6 OR of developing ALS compared to controls within 1 year (Roos et al., 2016). Likewise, a diagnosis of depression is more probable after ALS onset, in particular within 1 year from the appearance of motor symptoms (Roos et al., 2016; Turner et al., 2016). The same increased prevalence before and after ALS onset is true also for anxiety symptoms and neuroticism (Longinetti et al., 2017). Familial history of suicide was extremely overrepresented in ALS kindreds (Byrne et al., 2013; O’Brien et al., 2017).

On the other hand, many of the behavioral alterations demonstrated in ALS patients may be confused with depressive symptoms, such as apathy, which is found in 31–88% of patients (Witgert et al., 2010; Lillo et al., 2011), self-centredness, blunting of primary emotions, and lack of concern for personal hygiene (Gibbons et al., 2008). Interestingly, some studies failed to relate these symptoms to measures of depression (Grossman et al., 2007), arguing that this may reflect a behavioral disturbance due to the intrinsic ALS-FTD pathological continuum, rather than being secondary to the mood disorder. Another potential confounder of depressive symptoms in ALS is pseudobulbar affect (PBA), a neurobehavioral phenomenon manifesting with pathological overwhelming laughter or crying which are either incongruent with or excessive for the context. However, it has been demonstrated that crying-predominant PBA is associated with depression, while laughter-predominant is not (Thakore and Pioro, 2016). This finding may be due to mutual contributions from these conditions, with depression presenting as crying in the setting of PBA, and crying from PBA reinforcing the feeling of sadness and underlying depression. Further studies will be needed to determine whether these emotional phenomena in ALS are related to a shared pathological mechanism with depression.

### Autism, Obsessiveness and Other Psychiatric Disorders Associated With ALS

Among other psychiatric disorders associated with ALS, Turner et al. found increased rate of bipolar disorder, with a relative risk of 3.2 to develop ALS within 1 year from hospitalization (Turner et al., 2016). This was later confirmed by a large registry-based study in which bipolar, neurotic and stress-related disorders, as well as a history of drug abuse/dependence, represented risk factors for subsequently developing ALS (Longinetti et al., 2017). Clustering of autism spectrum disorders has been observed within ALS and c9orf72 positive ALS-FTD kindreds (O’Brien et al., 2017; Devenney et al., 2018). Lack of empathy is commonly reported among the cognitive deficits of ALS patients (Cerami et al., 2014). Other abnormalities typically associated to autism such as stereotypical behaviors, social cognition impairment, obsessive-compulsive traits and mental rigidity have been reported in patients and corresponds to the clinical ALS-FTD continuum (Gibbons et al., 2008; Lillo et al., 2010; Mioshi et al., 2014). Theory of Mind (ToM) refers to the ability to infer mental states of oneself and others such as beliefs, emotions, intentions, and desires, thus allowing for an understanding of other people’s behavior; these capacities are typically compromised in autistic patients (Hoogenhout and Malcolm-Smith, 2017). ToM processes have been further subdivided in cognitive and affective components. In ALS, 36% of patients displayed impairment in cognitive abilities, whereas 27% were dysfunctional in the affective ToM (van der Hulst et al., 2015).

Medial and orbitolateral prefrontal cortices have been involved in ToM capacity (Gallagher and Frith, 2003; Mitchell et al., 2006; Völlm et al., 2006), and several studies have largely demonstrated dysfunctional networks in these areas (Meier et al., 2010; Trojsi et al., 2017). Overall, a selective neurochemical or neuroanatomical network disruption may lie beneath ALS and autism mediated by unknown pathological mechanisms that warrant further research.

## Histopathological Signature of the ALS-FTD Spectrum and Hints for Correlations to Psychiatric Symptoms

The most common histopathological feature in ALS is represented by TDP-43 inclusions in motor neurons, either large and round (Lewy bodies-like) or skein-like (Braak et al., 2010). TDP-43 can be mislocalized within neuronal cytoplasmatic inclusion (NCIs) or dystrophic neurites (DN), and is enriched in post-translational modifications such as ubiquitination and phosphorylation (Tan et al., 2013).

Larger works proved TDP-43 pathology to be present in about half of all FTD cases (Davidson et al., 2007; Mann and Snowden, 2017), whereas the remnant 45% is represented by protein tau, and less than 5% by FUS or other aggregate-prone proteins (Mann and Snowden, 2017).

Interestingly, based on morphology and distribution of the inclusions, TDP-43 pathology in ALS and FTD can be subclassified into four types (A, B, C, D), the first two of them displaying round intracytoplasmatic aggregates (Tan et al., 2013). Type B is the most typically observed inclusion pattern in MND, while in FTD it is observed only in patients showing concomitant motor neuron involvement (Burrell et al., 2016).

Propagation of TDP-43 pathology from its core anatomical substrate, i.e., the motor cortex, was shown in ALS from a large cross-sectional autopsy study in which four stages of progression were identified. In stage 1, TDP-43 proteinopathy can be observed in the granular motor neocortex, alpha-motoneurons of the ventral horn, and bulbar motor neurons of cranial nerves. Stage 2 is characterized by involvement of reticular formation and precerebellar nuclei. In stage 3 TDP-43 inclusions are present in the prefrontal neocortex (firstly, gyrus rectus and orbital gyri, and, secondly, sensory areas and temporal neocortical area) and basal ganglia (striatum and inferior colliculus). In the final stage (4), anteromedial areas of the temporal lobe and the hippocampal formation display signs of pathology (Brettschneider et al., 2013).

A similar mechanism of spreading has been observed for bvFTD, where involvement of the orbital gyri, gyrus rectus, and amygdala characterizes the cases at the very initial phase (stage I). At an increasing burden of disease, the middle frontal and anterior cingulate gyrus as well as anteromedial temporal lobe areas, superior and medial temporal gyri, striatum, red nucleus, thalamus, and precerebellar nuclei are involved (stage II). More advanced phases of disease are characterized by motor cortex, bulbar somatomotor neurons, and spinal cord anterior horn propagation (stage III), and ultimately, visual cortex is affected (stage IV) (Brettschneider et al., 2014).

With regard to psychosis and schizophrenia, though they are considered more as diseases of connectivity and abnormal neurochemical transmission, some histopathological studies have found small but significant areas of atrophy in hippocampus, prefrontal and superior temporal cortex, and thalamus. This is accompanied by hemispheric asymmetry, decreased cortical thickness and gyrification, and abnormalities in hippocampal shape. Moreover, an early neurodevelopmental anomaly in schizophrenia may be postulated since the discovery of abnormally placed and clustered neurons in lamina II of entorhinal cortex or in the neocortex (Harrison and Weinberger, 2005). The finding of a decreased number of dendrites and arborization at hippocampal and neocortical level further support the view of reduced or aberrant wiring in schizophrenic patients, while the major neurochemical findings at cortical levels are represented by reduced number of serotoninergic (5-HT2A) and muscarinic (M1) receptors in patients with schizophrenia (Dean et al., 2016).

A clear neuropathological hallmark similar to the ALS/FTD spectrum does not exist for schizophrenia; nevertheless, in an autopsy case series on schizophrenic patients, tau-positive glial tangles were found in the dorsal aspect of temporal horn, arcuate fibers in gyri of frontal cortex, and within parahippocampal gyrus, while neurofibrillary tangles were observed in transentorhinal cortex, entorhinal region, subiculum and anterior hippocampus, in almost one third of cases, with increasing prevalence in elderly patients (Casanova et al., 2002). Altogether, these data may point to a restricted limbic tauopathy in presenile or senile psychotic patients, with no evidence of progression unlike Alzheimer disease. Though it may not be excluded that aberrancies in tau metabolism are due to neuroleptic drugs (Wisniewski et al., 1994), it is reasonable to hypothesize neurodegenerative processes occur in schizophrenia as well, given the accelerated aging and overall atrophy resulting in severe cognitive decline and motor abnormalities from duration of untreated psychosis (Anderson et al., 2014).

A further neuropathological link between psychosis and ALS may be found in microglia activation.

In fact, ALS arises in part by non-cell-autonomous mechanisms, from a combination of damage within MNs and their glial partners (Boillée et al., 2006). During the disease course, microglia switches from a neuroprotective M2 phenotype to an activated M1 phenotype which secretes proinflammatory interleukines, cytokines and neurotoxic factors, leading to the progression of neuronal injury (Henkel et al., 2013). Similarly, in schizophrenic patients’ autopsies, increased markers of microglia activation were observed in the prefrontal cortex, anterior cingulate and temporal cortex (Radewicz et al., 2000).

## Neuroimaging Across Psychiatric and Behavioral Symptoms in ALS

Neuroimaging studies have been crucial to better investigate functional and structural alterations in ALS patients showing psychiatric symptoms. By studying brain volume in a cohort of bvFTD and FTD-MND patients, a precise network of cortical and subcortical areas could be identified in patients with psychotic symptoms, which display bilateral medial prefrontal and occipital cortices, right thalamus and left cerebellum atrophy (Devenney et al., 2016). Sub-analysis within c9orf72 expansion carriers with psychosis prior to FTD or FTD-MND revealed that, besides presenting higher psychotic index, more extensive network disruption occurred, with volume reduction of bilateral medial frontal cortex, anterior cingulate and orbitofrontal cortex, bilateral insula, caudate, putamen and thalamic nuclei, middle, inferior and superior temporal gyrus, temporal fusiform gyrus, lateral occipital cortex and right cerebellum (Devenney et al., 2016). These areas roughly corresponds to those with the highest degree of atrophy in schizophrenic and schizoaffective patients (Amann et al., 2016). Moreover, anterior cingulate cortex and insula are strongly connected in the salience network, whose main function is to detect, analyze and integrate emotionally salient stimuli with respect to the internal environment, and which is involved in symptom generation in both FTD and schizophrenia (Seeley et al., 2007; Zhou and Seeley, 2014). In addition to schizophrenia, late-onset obsessive compulsive disorder in the setting of an upper motor neuron disease with concomitant FTD presents with bilateral hippocampal atrophy with sclerosis of right hippocampus on MRI and moderate right temporal cortex thinning at PET imaging (Bersano et al., 2018).

As already mentioned, abnormal behaviors are found in ALS patients along the FTD spectrum. Among these, apathy is one of the most commonly reported, and is correlated with cortical thickness reduction in bilateral orbitofrontal lobe and left precentral gyrus. On the other hand, a hostile, disinhibited pattern of personality as identified by PCA analysis is more associated to thinning of temporal and cingular regions of the right hemisphere (Consonni et al., 2018).

## A Common Genetic Background

Genome wide association studies (GWAS) allowed for exploring the genetic relationship between schizophrenia and ALS through SNPs-based heritability estimates, obtaining a genetic correlation of 14% (McLaughlin et al., 2017) due to polygenic overlap. Intriguingly, conditional false discovery rate was used to investigate novel ALS-associated genomic loci, confirming some of the known pleiotropic risk loci discussed below. A further study found clustering of schizophrenia and psychosis, suicide, autism, rigid personality disorders, and alcoholism in ALS kindreds suggesting that shared pleiotropic oligogenic variants may be responsible for co-segregation of psychiatric illnesses and ALS (O’Brien et al., 2017).

### c9orf72

In 2011 a worldwide effort identified a hexanucleotidic expansion in the c9orf72 gene as the major genetic determinant of both ALS and FTD (DeJesus-Hernandez et al., 2011; Renton et al., 2011), thus revolutionizing our knowledge of genetic pleiotropy of ALS. C9orf72 pathological expansion accounts for almost 40% of fALS, 8% of sALS, and almost 30% of familial FTD in Caucasian population (Ng et al., 2015; Ng and Tan, 2017). This prevalence is increased in ALS-FTD, where it is found in 50–70% of familial and 15–20% of apparently sporadic cases (van der Zee et al., 2013). Noteworthy, c9orf72 repeat expansion display a high phenotypic variability, spanning from parkinsonism (Floris et al., 2012), to corticobasal degeneration (Lindquist et al., 2013), psychosis (Watson et al., 2016), and suicidal behavior (Synofzik et al., 2012). Penetrance is incomplete and age-dependent (Murphy et al., 2017), with anticipation phenomena similar to other repeat expansions diseases (Van Mossevelde et al., 2017). When analyzing the clinical feature best discriminating c9orf72 carriers from non-carriers in a cohort of bvFTD patients, psychosis and familiality for ALS appeared the most reliable clues (Devenney et al., 2014). A recent study investigating the risk of psychiatric disorders in c9orf72 positive kindreds, extrapolated from FTD and ALS cohorts, revealed an association with increased risk of autism spectrum disorders (HR: 2.7), schizophrenia (HR for a family member: 4.9) or a single psychotic episode (HR: 17.9), and mood disorder (HR: 1.9) (Devenney et al., 2018). Overall, this study confirms previous reports from an aggregation study in which stratification of ALS probands in carriers and non-carriers of c9orf72 repeat expansion was associated with major risk of presenting psychiatric disturbances in family members (Byrne et al., 2013). Importantly, among the referred psychiatric disorders associated to c9orf72 expansion, obsessive-compulsive disorder seems to be excluded (Arthur et al., 2017), though rigid stereotyped behavior with obsessiveness is frequently observed in carriers (Snowden et al., 2012).

C9orf72 is an alternatively spliced gene encoding for three protein transcripts, whose functions have not been fully elucidated. Molecular studies showed that the protein localizes in the nucleus and is structurally similar to DENN (differentially expressed in normal and neoplasia) proteins, which contain a guanine nucleotide exchange factor allowing them to interact with RAB GTPase proteins and regulate membrane trafficking from the nucleus (Levine et al., 2013; Aoki et al., 2017). A striking characteristic of c9orf72 alterations is that differential repeat length is observed across different tissues (Van Blitterswijk et al., 2013), suggesting instability and possibly the occurrence of epigenetic phenomena such as hypermethylation as a potential source of this variability (Xi et al., 2014).

Healthy individuals carry up to 25 repeats of GGGGCC in c9orf72, with the majority having a couple of repeats, while in ALS and FTD cases the number of repeats ranges from 100s to 1000s (DeJesus-Hernandez et al., 2011; Renton et al., 2011; Beck et al., 2013). Uncertainty surrounds the role of intermediate length (22–30) repeats, though they seem to be associated with a higher frequency of psychiatric symptoms in FTD, FTD-ALS, and atypical parkinsonism cohorts (Ng and Tan, 2017).

Recently some studies focused on biomarkers that may help predict the so-called “phenoconversion,” since genetic therapy may become an option for in C9orf72 carriers (Floeter and Gendron, 2018). Biological markers might guide pharmacological response to potential therapies, as in the case of Poly(GP) proteins (Gendron et al., 2017a), or predict the prognosis (Gendron et al., 2017b) and anticipate the onset of symptoms by a year (Benatar et al., 2018), as in the case of neurofilament heavy and light chain, respectively. Furthermore, imaging studies showed that atrophy of several cortical and subcortical structures have been observed in asymptomatic carriers, including the thalamus (Papma et al., 2017; Bertrand et al., 2018; Floeter and Gendron, 2018), the left caudate and putamen, besides diffuse cortical thinning in defined temporal, parietal, and occipital regions (Walhout et al., 2015). Similarly, white matter tracts are not spared either before symptoms onset: functional studies have shown salience and medial pulvinar networks, who are known connectivity networks prominently affected in bvFTD, to be severely disrupted in carriers already in their 40s (Lee et al., 2016). Increased radial diffusivity has been reported as well in the right anterior thalamic radiation and the right forceps, even in younger c9orf72 expansion carriers (Bertrand et al., 2018). Notwithstanding, asymptomatic carriers did not show significant atrophy before symptoms onset in a longitudinal voxel-based morphometry study (Floeter et al., 2016). When testing the hypothesis that psychiatric disturbances might be prodromal of the structural and functional brain abnormalities observed in c9orf72 presymptomatic carriers, Lee et al. (2016) found that carriers and non-carrier family members had comparable lifetime histories of psychiatric symptoms, non-carriers family members doubled the amount of psychiatric medications compared to carriers, and underwent similar rates of hospitalization for psychiatric disturbances. Overall, we cannot exclude that familiality for psychiatric diseases, which are known polygenic conditions, runs independently of the expansion among c9orf72-families, however, further studies are warranted to better explore prodromal disturbances of thought in expansion carriers because of the high variability between personality and behavioral tests, which might not be suited to detect subtle changes in non-demented cohorts.

### Other Genes Associated With Psychiatric Disturbances

Notwithstanding the major role of c9orf72, a non-trivial residual association between ALS and psychiatric disorders persists even after excluding repeat expansion carriers from genetic analyses (Byrne et al., 2013). Isolated cases of concomitant psychiatric disorders such as schizophrenia have been found in kindreds with specific mutations in FUS (Yan et al., 2010) and TARDBP (Quadri et al., 2011). Variable rates of psychiatric illnesses, generally less common than in c9orf72 repeat carriers, were also observed in non-c9orf72 ALS-FTD cases, carrying PRGN (Hall and Finger, 2015), TBK1 (Van Mossevelde et al., 2016), and VCP (Weihl, 2011) mutation. ATXN2, has also been associated to both ALS and schizophrenia risk (Zhang et al., 2014).

It is reasonable to speculate that the numerous genetic loci known to be involved in the ALS-FTD disease spectrum, such as TBK1, PGRN, CHCHD10, TUB4A, VCP, may predispose to psychiatric illnesses by analogous mechanisms to c9orf72. The rarity of these cases, together with the relatively small populations studied, and the difficulty in discerning psychiatric disturbances from other aspects of behavioral FTD, make proving this assumption a daunting task.

## Prognostic Role of Psychiatric Disturbances in ALS

Concomitant psychiatric diseases in ALS patients, whether prior or after this fatal diagnosis, may add strain on caregivers and pose important ethical challenges for support and end-of-life decisions along the course of this disease. Until now, only few register-based studies have taken into account the prognostic significance of simultaneous psychiatric illness in ALS, showing a mild negative influence of anxiety symptoms and other psychiatric disturbances in univariate analysis, whose effect was later unconfirmed in multivariate analysis (Körner et al., 2013; Mandrioli et al., 2018). Other studies examining the impact of neuropsychiatric symptoms in ALS, expressed mainly as behavioral alterations, failed to demonstrate any impact on survival (Mioshi et al., 2014; Burke et al., 2017). However, in a prospective study evaluating depression in ALS by validated outcome measures, a concurrent diagnosis of major depressive disorder corresponded to decreased survival, and any increasing score matched increased death hazard ratio (Thakore and Pioro, 2016). This discordance in findings may be related to an underestimation of depression in the ALS population. In addition, the use of more subtle evaluating psychometric measures may aid the clinician to formulate such a diagnosis and address these disorders, which are increasingly reported as a major burden for carers (Creemers et al., 2016).

## Conclusion

In conclusion, psychiatric diseases often anticipate the onset of motor symptoms in ALS, and their timely relation with motor neuron pathology may be due to underlying common pathogenic mechanisms affecting non-motor structures within the central nervous system. Similar changes in structural framework between ALS, ALS-FTD and schizophrenia exist, and some degree of genetic overlap between these diseases has been found, strengthening a common pathological signature. Overall, psychiatric illness do not appear to influence significantly the prognosis and survival of ALS patients, but may constitute an increased burden for caregivers and challenge ethical choices with regards to end-of-life decisions. Thus, clinicians should be aware of the tight relationship between ALS and psychiatric disorders and timely address specialist interventions to better assist ALS families.

## Author Contributions

JM, EZ, and NT contributed to conceptualization, data curation, formal analysis, and methodology. EZ wrote the first draft. and JM and NT reviewed and edited it.

## Conflict of Interest Statement

The authors declare that the research was conducted in the absence of any commercial or financial relationships that could be construed as a potential conflict of interest.
